# Predicting Students' Attitudes Toward Collaboration: Evidence From Structural Equation Model Trees and Forests

**DOI:** 10.3389/fpsyg.2021.604291

**Published:** 2021-03-26

**Authors:** Jialing Li, Minqiang Zhang, Yixing Li, Feifei Huang, Wei Shao

**Affiliations:** ^1^School of Psychology, South China Normal University, Guangzhou, China; ^2^Key Laboratory of Brain, Cognition and Education Sciences (South China Normal University), Ministry of Education, Guangzhou, China; ^3^Center for Studies of Psychological Application, South China Normal University, Guangzhou, China; ^4^Guangdong Key Laboratory of Mental Health and Cognitive Science, South China Normal University, Guangzhou, China

**Keywords:** attitudes toward collaboration, person-centered, most significant factors, PISA 2015, structural equation model tree, structural equation model forest, data mining

## Abstract

Numerous studies have shed some light on the importance of associated factors of collaborative attitudes. However, most previous studies aimed to explore the influence of these factors in isolation. With the strategy of data-driven decision making, the current study applied two data mining methods to elucidate the most significant factors of students' attitudes toward collaboration and group students to draw a concise model, which is beneficial for educators to focus on key factors and make effective interventions at a lower cost. Structural equation model trees (SEM trees) and structural equation model forests (SEM forests) were applied to the Program for International Student Assessment 2015 dataset (a total of 9,769 15-year-old students from China). By establishing the most important predictors and the splitting rules, these methods constructed multigroup common factor models of collaborative attitudes. The SEM trees showed that home educational resources (split by “above-average or not”), home possessions (split by “disadvantaged or not”), mother's education (split by “below high school or not”), and gender (split by “male or female”) were the most important predictors among the demographic variables, drawing a 5-group model. Among all the predictors, achievement motivation (split by “above-average or not”) and sense of belonging at school (split by “above-average or not” and “disadvantaged or not”) were the most important, drawing a 6-group model. The SEM forest findings proved the relative importance of these variables. This paper discusses various interpretations of these results and their implications for educators to formulate corresponding interventions. Methodologically, this research provides a data mining approach to discover important information from large-scale educational data, which might be a complementary approach to enhance data-driven decision making in education.

## Introduction

Collaboration is one of the most important twenty-first-century skills. Increased demands are placed on citizens' ability to collaborate with others. Collaboration is embedded in the fabric of daily life. A series of studies revealed that collaboration could enhance team cohesion, generate positive evaluations of partners, and improve performance (Mishra et al., 2015; Chen and Agrawal, 2017; Aldieri et al., 2018). To better improve people's collaborative performance, it is worth noting that collaboration involves much more than physically gathering together to discuss issues or share information among team participants. It also relies heavily on team members' involvement, attitude, and commitment regarding interacting with each other (Wu et al., 2013). Team members' attitudes toward collaboration are important for successful collaboration (OECD, 2017a).

Attitude toward teamwork is defined as the individual willingness to continue working together with the same team as well as in other teams (Gardner and Korth, 1998). In Gardner and Korth's (1998) study, students' attitude toward teamwork was measured by seven items, 2 for students' enjoyment in group work and 5 for the benefits in group work. Mendo et al. (2017) developed a measurement instrument for attitudes toward teamwork with two distinct factors: academic attitudes related to students' learning outcome and individual success and social attitudes related to students' appraisal of the interaction when working with others. Most previous studies agreed with the opinion that the structure of collaborative attitudes should include two components: intellectual benefit and social relationship.

John Dewey identified schools as the ideal environment for students to learn about interconnecting and internalize the importance of cooperation (Dewey, 1916). School years serve as a crucial period for students' development of collaboration, making it necessary for educators to explore effective policies to improve students' attitudes toward collaboration. Since a positive attitude is essential for collaboration, a growing body of educational literature has identified numerous predictors of students' attitudes toward collaboration. Those studies explored the influence of factors in isolation specifically, which are essential to reveal the underlying mechanism and develop psychological theory. However, it has been difficult to compare the results of such separate studies with each other. In practice, educators are hard to identify the most important factors from various ones related to students' attitudes toward collaboration.

Since education is a complex system and educational data cover various aspects of student development, data-driven decision making (DDDM) is attaching the attention of educators. A growing body of research considers how educators can effectively use data collection and analysis to drive practice (Halverson et al., 2007; Mandinach, 2012). The development of information technology provides a convenient way for educators to collect data on students' development, while the data mining techniques improve the use of big data in education (Sin and Muthu, 2015). For example, Sorensen (2019) used machine learning techniques incorporating 74 predictors about academic achievement, behavioral indicators, and socioeconomic and demographic characteristics to identify student dropout risk. In the context of DDDM, the current study used data mining techniques to analyze a large scale dataset about various aspects of students, aiming to identify which factors are most important for students' attitudes toward collaboration and reveal their interactions. For educators, data mining from a large number of potential predictors may provide a complementary way, which is beneficial for educators to focus on key factors and make effective interventions at a lower cost (Sorensen, 2019).

In the first part of the introduction, we reviewed the literature on several types of factors that affect students' attitudes toward collaboration. In the second part, we introduced the PISA 2015 program, the first large-scale test of students' collaborative ability and attitudes (OECD, 2017a). Besides, the PISA dataset provided a number of students' features proven to be associated with students' collaborative attitudes, allowing researchers to compare and dig out the most important key factors. The third part introduced two data-mining methods: structural equation model trees (SEM trees; Brandmaier et al., 2013) and structural equation model forest (SEM forests; Brandmaier et al., 2016). These methods are effective tools to compare multiple factors and their interactions simultaneously. In the last part, we claimed the main goals of this study.

### Variables Related to Students' Attitudes Toward Collaboration

Researchers do know a fair amount already about which individual factors contribute to students' attitudes toward collaboration. Generally, these studies examined the relationships between specific factors and students' attitudes, aiming to enhance the understanding of the underlying mechanism. Here, we introduce three types of variables in terms of their relationships to the individual, i.e., demographic variables, personal traits, and students' activities.

Generally, the most widely explored are demographic variables, as they are relevant to questions about educational opportunity and equalities. For example, girls were found to be more willing to show a cooperative attitude than boys, while boys cooperated less often to signal that they were tough when observed by their peer group (Carlo et al., 2007; Charness and Rustichini, 2011). Possible interpretations were gender intensification and differential socialization pressures (Fabes et al., 1999). Findings related to the influence of age were contradictory, which might be due to differences in methodology (Jackson and Tisak, 2001). As for the family background, family resources are positively associated with students' prosocial tendency and attitudes toward others. Individuals with adequate family resources during childhood have stable interpersonal bonds, enjoy cooperation, and are more likely to trust and act altruistically (Stamos et al., 2019). Li et al. (2019) found that students with a higher childhood socioeconomic status (SES) exhibited higher altruistic intentions and behaviors when facing or imagining a threat scenario in three experimental environments. Having lower financial conditions in childhood was associated with lower mental health and well-being in adulthood. Lower mother's education was associated with lower well-being, while higher father's education was associated with lower well-being (Sheikh et al., 2016). This reflected the important role of mothers in the mental health of children.

Besides, students' personal traits influence how they perceive and react to the social world around them while in collaboration. Individuals with high levels of affiliation motivation or power and/or achievement motivation show a significantly high level of teamwork skills and team effectiveness (Yi and Park, 2015). Achievement goals influence people's information exchange. Mastery goals lead to an open and cooperative mind-set, while performance goals lead to a competitive mind-set and exploitation orientation (Poortvliet et al., 2007). Positive thinking has been reconfirmed to have a significant impact on collaborative dispositions for students. Individuals who highly value others are more willing to collaborate with team members (Wu et al., 2013). When students are able to develop mature communication, accountable interdependence, psychological safety, have a common purpose and clear understanding of their role, they are more likely to have a better attitude toward teamwork (Ruiz Ulloa and Adams, 2004).

Students' positive cooperation experiences and the time on teamwork could enhance students' attitudes toward collaboration. Pfaff and Huddleston (2003) assess numerous predictors of marketing student attitudes toward team projects. They found that project grades, perceived workload, time in class for teamwork, use of peer evaluations, and absence of a “free-rider” problem were significant predictors of attitudes toward teamwork. Leadership and group size appear to have no influence on student perception of teamwork. An educational experiment suggested that school-based dancing programs encouraging coordinated physical activity in student groups developed students' collaborative networks (Zander et al., 2014). Interprofessional curriculum or practice experience has been proven to successfully enhance students' attitudes regarding collaboration with other health care professionals (Curran et al., 2005; Park et al., 2014). Regarding off-class activities, a Facebook study showed that instrumental support from Facebook friends was a powerful factor predicting class-related academic collaboration (Khan et al., 2014). Collaborative video games can also help improve students' collaborative attitudes and performance (Nebel et al., 2017).

Given the growing attention to attitudes toward collaboration, research attempting to improve the prediction of attitudes toward collaboration has increased significantly in recent decades. However, the differences in methodology, measurement instruments, and samples generated different datasets and models, making the results not comparable to each other. The relative importance of these factors and their interactions is still unclear.

### PISA: A Large-Scale Educational Assessment

Organized by the Organization for Economic Co-operation and Development (OECD), the Program for International Student Assessment (PISA) is one of the largest global assessment programs in education. PISA assesses the key knowledge and skills essential for 15-year-old students to have acquired for full participation in modern society. In 2015, PISA focused on science, reading, mathematics, and collaborative problem-solving (CPS). Furthermore, students also answer a background questionnaire that seeks information about the students themselves, including questions about their homes as well as their school and learning experiences.

In PISA 2015, students' attitudes toward collaboration were measured in terms of two dimensions: valuing relationship and valuing teamwork. The four statements regarding valuing relationship were related to altruistic interactions, i.e., when the student engaged in collaborative activities that were not for his or her own benefit. On the other hand, the four statements regarding valuing teamwork were related to what teamwork could produce. These two dimensions were consistent with the previous structure of students' attitudes toward collaboration. In addition, as one of the largest global assessment programs in education, PISA also measures an extensive range of students' features to provide an in-depth analysis of education policies and practices. According to the PISA 2015 report on collaborative problem-solving, a number of students' features were proven to be associated with students' collaborative attitudes (OECD, 2017a).

First of all, differences between different demographic groups of students are popular topics in educational studies, which is relevant to questions about educational opportunity and equalities. On average across OECD countries, girls were significantly more likely than boys to value relationship, while boys tended to value teamwork more than girls. Generally, students in the top quarter of economic, social, and cultural status (ESCS) (advantaged students) reported higher scores in valuing relationship. However, students in the bottom quarter (disadvantaged students) were more likely to value teamwork. Since ESCS was derived from several variables, we were also concerned about these variables to explore the more detailed relationships between ESCS and students' attitudes toward collaboration. In addition to students' gender and ESCS, PISA 2015 provided individual variables related to school, such as grade repetition, school changes. Although these variables did not show a significant difference, we considered them to explore whether there are interactions between these variables and those significant variables.

In addition, PISA 2015 Results (OECD, 2017c) analyzed a variety of well-being indicators. Life satisfaction, achievement motivation, sense of belonging, and schoolwork-related anxiety at school were positively associated with attitudes toward collaboration. Exposure to bullying was negatively associated with. As for campus activities, students who participated in physical activity or interaction in science class more frequently were more positive in cooperation. Truancy harmed students' attitudes toward collaboration, either for the truant students or for his or her non-truant students. Regarding off-campus activities, students who used the telephone or Internet to do social activities outside school would prefer working as part of a team to working alone. The same conclusion could be found in students who are more involved in family affairs and play video games.

Since the PISA 2015 dataset was an open-source for secondary analysis and provided a large number of students' features as well as collaborative performance and attitudes, researchers have been able to expand new researches on CPS (Herborn et al., 2017; Sum and Bădescu, 2018; Stadler et al., 2020). However, the previous research, including the PISA report, did not test an integrated model and the relative importance of these related factors. As mentioned above, the predictors from PISA 2015 reflected individual factors across a number of domains. Such a typical database for DDDM allowed educators to identify important information, which was beneficial to economize educational resources and make effective interventions. In this study, we chose the variables mentioned above to enter the data mining model, for they have been previously shown to have a significant relationship with students' attitudes toward collaboration. All variables were derived according to the technical report of PISA 2015 (OECD, 2017b).

### Data-Mining Methods: SEM Trees and SEM Forests

In social and educational research area, data now are proliferating and new sources of data continue to emerge. Tree-based data mining techniques have begun to gain prominence in the social sciences (Hu et al., 2014; Brown et al., 2019; She et al., 2019). In the current study, two recently proposed data mining methods were used: SEM Trees and SEM Forests. SEM trees are the combination of decision trees (DTs) and the structural equation modeling framework (Brandmaier et al., 2013; Jacobucci et al., 2016). As DT's extension, an SEM tree groups participants recursively into subgroups that are maximally different with respect to the fit of the hypothesized and template SEM (e.g., path model, factor analysis model, and longitudinal growth curve model). Instead of fitting a single SEM to the data, the data set is partitioned into subsets based on the splitting of covariates and SEMs are fit to each subset. Moreover, Brandmaier et al. (2016) extended SEM trees to an SEM forest, assembling hundreds or thousands of trees and aggregating the predictions of the individual trees. An SEM forest provides a stable statistic, namely variable importance, that presents the extent to which variables predicted differences according to the template model. Generally, there are two main applications of tree-based models: (1) variable selection and (2) sample classification.

As for variable selection, the most common advantage of tree model is the automatic search for non-linear effects and higher-order interactions. In classical statistical models, including linear, and logistic regression as the most popular representatives of standard parametric models, the functional form of association pattern is restricted apriori and interaction effects of high order are included manually. Such characteristics make it limited when analyzing the high dimension data (Strobl et al., 2009; Gonzalez et al., 2018). As a model-based tree technique, SEM trees automatically search the most informative splits and generates the interaction between factors (Merkle and Shaffer, 2011). It does not assume a functional form between covariates and dependent variables. With limited information about the influence of high dimension factors and their interactions, SEM trees might be a preferable approach that could identify the most important variables and their interaction in a data-driven way (Jacobucci et al., 2016; Usami et al., 2017). The tree structure provides interpretable and visual rules about predictive information. The different levels of splitting variables represent the relative importance and their interactions. When the independent variables are mostly categorical variables or ordered variables, e.g., demographic variables, their interactions are often the focus of studies.

From the aspect of sample classification, SEM tree is a person-centered approach that provide interpretable rules for classification. The result of SEM trees could be described as a classification tree structure, which maps onto the way that participants are grouped into different subgroups based on a series of conditional (Boolean) rules. Each subgroup of SEM tree represents an SEM structure with a distinct set of parameter estimates that significantly different from other subgroups. Due to the classification function, SEM trees serve as an important approach to uncover the potential population heterogeneity. In traditional cluster models, such as cluster analysis (Fraley and Raftery, 1998) and finite mixture models (FMM; Lubke and Muthén, 2005), heterogeneity is manifested by allowing for unobserved groups or latent classes. When researchers are more interested in the sources of heterogeneity and make corresponding interventions, SEM trees, exploring heterogeneity by grouping samples according to observed covariates, might be a more intuitive approach for educators. It can be considered as exploratory multigroup SEMs hierarchically.

Due to the flexibility of the SEM framework and data mining, SEM trees and forests have attracted empirical researchers' attention (de Mooij et al., 2018; Ammerman et al., 2019, 2020; Fuhrmann et al., 2019; Casanova et al., 2020; Simpson-Kent et al., 2020; Serang et al., 2021). For example, Ammerman et al. (2020) used 46 indicators to construct a four-factor model as the template model of SEM trees, assessing borderline personality symptomology, childhood maltreatment, suicidal thoughts and behaviors, and depressive symptomology. Utilizing SEM trees and SEM forests, they identified five subgroups of different self-injury severity based on non-suicidal self-injury frequency and/or methods. In the current study, we measured students' attitudes toward collaboration by conducting a factor analysis model as the template model. Since PISA provided a large number of students' features that educators concerned about, SEM trees and forests offered us an effective tool to explore the most informative predictors and different subgroups.

### The Current Study

Research that considers the relative importance of influence factors and their combinations in relation to students' attitudes toward collaboration is absent from the literature. And the utility of SEM trees and forests for DDDM has not been illustrated. The current study aimed to (1) explore the most significant factors and their interactions from a large number of covariates; (2) group students in order to draw a concise model; and (3) help researchers and policymakers formulate specific and effective interventions for different groups of students. To address these aims, SEM trees and forests were applied to examine the interplay between students' attitudes toward collaboration and related variables in a comprehensive large-scale education database, i.e., PISA 2015 dataset.

There are two main contributions of this study. The first contribution is empirical. Exploring various factors about students, including students' family background, personal traits, and behaviors, we extended the existing research to integrated model for educators with a national dataset. The results from the current study will improve our understanding of the important predictors of students' attitudes toward collaboration, which is beneficial to help educators focus on these key factors and develop appropriate measures for different types of students at lower costs. The second contribution is methodological. With the DDDM strategy, two data mining techniques were applied in educational data. SEM trees and forests, combining the SEM framework and data mining techniques, offer great flexibility in modeling relationships between indicators and variables. This study illustrated how SEM trees and forests help educators make good use of educational data. Although empirical education research has benefitted greatly from the development and improvement of quasi-experimental methods, developments in the areas of data science and computational methods may provide a complementary approach for educators to effectively use indicators recorded during educational process.

## Materials and Methods

### Dataset From China

Cross-country comparisons of attitudes toward collaboration are difficult to interpret given the cultural differences between countries and economies (OECD, 2017a). However, subgroups in each country/economy may respond differently. In this study, we only chose the dataset from China. Education has been considered an important pillar of national and social progress. The Chinese government attaches great importance to improve the quality of basic education and the all-around development of students (China, 2016). In PISA 2015, China did a good performance in science, reading and mathematics. However, in the collaborative problem-solving assessment, China performed below what would be expected given the performance in these academic assessments (OECD, 2017a). With respect to the students' questionnaire of attitudes toward collaboration, China ranked fourth among all OECD and partner countries and economies for valuing teamwork while only at the average level for valuing relationship. Therefore, it's important for educators and policy makers in China to pay more attention to improving students' collaborative abilities and attitudes.

This study used the PISA 2015 B-S-J-G (China) student questionnaire dataset, representing four PISA-participating provinces: Beijing, Shanghai, Jiangsu, and Guangdong. A total of 9841 students from 268 schools were selected to participate in the PISA 2015 assessment. Seventy-two cases with less than one response in each dimension of attitudes toward collaboration were discarded. Ultimately, the data consisted of 9769 cases (4649 females and 5120 males). According to the PISA 2015 report, differences across schools accounted for <3% of the differences in two indices of students' attitudes toward collaboration. Therefore, student-level variation explained most of the observed differences in attitudes toward collaboration. As such, this study specifically focused on exploring the relationships between the students' collaborative attitudes and a set of focus variables selected from the PISA student questionnaire, not including the school-level factors.

### Measurements

The measures adopted in the present study were taken from the PISA 2015 student questionnaires. The questionnaires included two types of variables: (1) single item (e.g., gender) and (2) derived variables based on two or more items (e.g., test anxiety). The single items were analyzed directly as categorical. However, individual participant scores of derived variables were estimated by weighted likelihood estimates (WLEs) and standardized to an international metric with an OECD mean of zero and an OECD standard deviation of one, which made it meaningless to subgroup students by WLE scores (OECD, 2017b). Moreover, PISA defined “advantage” as those in the top quarter of the distribution of the index and “disadvantage” as those in the bottom quarter (OECD, 2016). Therefore, the derived variables were discretized into categorical variables, partitioned by the 25th, 50th, and 75th percentiles, in order to investigate the influence of students' advantage or disadvantage in each aspect, rather than using a specific cut-off. Specifically, for each variable, students ranking in the top quarter were assigned to the category “4” (advantaged), while those in the second quarter were assigned to the category “3” (slightly advantaged), those in the third quarter to the category “2” (slightly disadvantaged), and those in the bottom quarter to the category “1” (disadvantaged).

In this study, independent variables were intentionally classified into two primary domains: demographic variables and variables of other attitudes and activities. This was based on the following considerations: (1) demographic characteristics of students are easily available for educators, which means such variables are the most fundamental to some extent; (2) other attitudes and activities of students may reflect their deeper traits and may therefore be more predictive, but they are not convenient to obtain; and (3) mixing the two types of characteristics may cause demographic characteristics to appear less salient (Sorensen, 2019). Therefore, we analyzed the two types of datasets separately: (1) dataset with demographic variables and (2) dataset with demographic variables as well as variables of other attitudes and activities (i.e., the whole dataset).

#### Dependent Variables: Attitudes Toward Collaboration

PISA 2015 measured students' attitudes toward collaboration in terms of two dimensions: valuing relationship (four items) and valuing teamwork (four items). It asked students about their agreement with specific cooperative aspects on a four-point Likert scale with the answers: “strongly agree,” “agree,” “disagree,” and “strongly disagree.” The index of valuing relationship (Cronbach's alpha index of internal consistency, α = 0.677) was related to altruistic interactions, meaning that students tend to be more interested in collaborative activities that are not for their own benefit. The index of valuing teamwork (α = 0.821) was related to the results of teamwork, meaning that students tend to see the benefits of teamwork.

#### Independent Variables: Demographic Variables

A rich set of demographic variables was available in the PISA dataset, including the following 17 variables: student international grade (D1), students' birth month (D2), students' gender (D3), school type (D4), mother's education (D5), father's education (D6), highest education of parents (D7), grade repetition (D8), school changes (D9), changes in educational biography (D10), highest parental education in years of schooling (D11), cultural possessions at home (D12, α = 0.658), home educational resources (D13, α = 0.650), home possessions (D14, α = 0.868), information and communication technology resources (D15, α = 0.713), family wealth (D16, α = 0.814), and ESCS (D17, α = 0.740).

#### Independent Variables: Other Attitudes and Activities

According to the results of PISA 2015 and previous studies, 16 attitudes and activities proven to be associated with students' attitudes toward collaboration were selected for data analysis. The variables associated with subjective well-being included six subscales: life satisfaction (F1), schoolwork-related anxiety (F2, α = 0.824), achievement motivation (F3, α = 0.780), sense of belonging at school (F4, α = 0.792), exposure to bullying (F5, α = 0.820), and teacher fairness (F6, α = 0.805). The variables associated with physical exercise were moderate physical activities (F7) and vigorous physical activities (F8) according to the exercise mode and time. Variables associated with off-campus activities were as follows: accessing the Internet/chat/social networks (F9), playing video games (F10), meeting friends/talking to friends on the phone (F11), and working in the household/taking care of other family members (F12). Three forms of truancy were skipping a whole day of school (F13), skipping some classes (F14), and arriving late for school (F15). Finally, student interaction in science class (F16, α = 0.898) was considered effective. Variables without Cronbach's alpha index of internal consistency were single-item variables.

### Data Analysis

#### Confirmatory Factor Analysis

Prior to data mining analyses, confirmatory factor analysis (CFA) was applied to determine the template model of an SEM tree. By constructing the latent factors, CFA provides measurements separated from measurement errors, offering greater validity. Higher mean scores on each latent variable indicate a more positive attitude toward collaboration. We assessed the overall fit of our models to the data using the chi-square test, root mean square error of approximation (RMSEA), comparative fit index (CFI), and standardized root mean square residual (SRMR). A good absolute fit was defined as RMSEA < 0.05, CFI > 0.97, and SRMR < 0.05, and an acceptable fit was defined as RMSEA = 0.08–0.05, CFI = 0.95–0.97, and SRMR = 0.05–0.10 (Schermelleh-Engel et al., 2003).

#### SEM Trees

By splitting observations into different groups according to their predictor values, SEM trees can be considered as a form of exploratory multiple group modeling. The resultant tree structure of an SEM tree represents several SEMs, each of which has a distinct set of parameter estimates. Observations were used in a confirmatory factor analysis (CFA), allowing us to examine group differences based on the CFA model parameters. We grew two types of SEM trees: (1) a demographic SEM tree with only demographic variables as the candidate predictors, and (2) a complete SEM tree with the variables of other attitudes and activities as well as the demographic variables.

Due to the characteristics of data mining methods, several hyperparameters need to be set in the program. We set the splitting method as *fair3*, which attempted to equate the predictor variables with the number of response values and retest all the splits with a holdout partition dataset. A Bonferroni correction was applied to control for Type I error. To prevent the model from growing too complex (a too-large tree), the *max depth* was set to 3 and the *minimum number of cases per node* was set to 500 (5% of the whole sample). Moreover, in order to examine group differences in the means of latent factors under the level of scalar invariance (also known as strong invariance), we constrained intercepts of the indicators and factor loadings to be the same across groups. According to previous studies of multigroup SEMs, scalar invariance is considered to be sufficient for the comparison of subgroups, ensuring the meaning and scale of each latent factor stayed consistent across subgroups (Clark et al., 2013).

#### SEM Forests

SEM forests randomly sample the cases uniformly and with replacements (bootstrapping), sample predictors without replacements for each tree, and make predictions. By averaging the decrease in fitness (i.e., log-likelihood) across all trees of the forest on the out-of-bag samples, an estimate of importance is obtained for each variable. Correspondingly, we grew two types of SEM forests: (1) a demographic SEM forest with only demographic variables as the candidate predictors, and (2) a complete SEM forest with the variables of other attitudes and activities as well as the demographic variables.

Also, several hyperparameters need to be set in the program. SEM forests were built with 500 trees in each forest. Each forest subsampled the cases in the bootstrap method and sampled predictors without replacement. The number of candidate predictors in the demographic SEM forest was $\sqrt{17}$, as there were 17 demographic variables. The number of candidate predictors in the complete SEM forest was $\sqrt{33}$, as there were 33 variables in total.

All analyses were conducted in R (R Core Team, 2013). The confirmatory factor analysis utilized full information maximum likelihood with the *OpenMx* package (Boker et al., 2011), and the SEM trees and SEM forests utilized the *semtree* package (Brandmaier, 2015). Additionally, in order to overcome problems with using the likelihood ratio test as the sole comparison of models and stopping criterion, we conducted multiple group analyses based on the result of SEM trees, using the Akaike information criterion (AIC; Akaike, 1973), Bayesian information criterion (BIC; Schwarz, 1978), and sample-size-adjusted BIC (aBIC; Sclove, 1987) to compare the different split level of the SEM models. Therefore, the false positive splitting could be pruned back. Moreover, *post-hoc* analyses examined differences across SEM trees and derived subgroups of indicators of collaborative attitudes to further probe the demonstrated SEM tree splits. Bonferroni *post-hoc* testing was used for multiple comparisons.

## Results

### Confirmatory Factor Analysis

**Figure 1:**
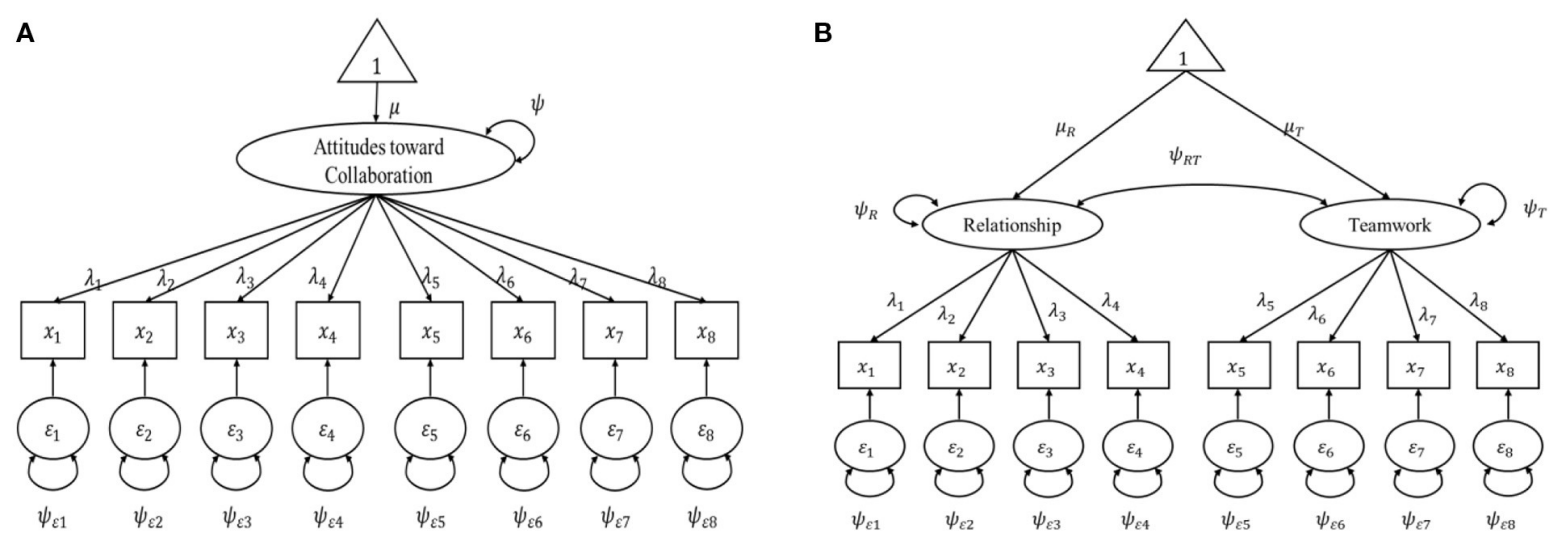
Factor model for students' attitudes toward collaboration. **(A)** One-factor model. **(B)** Two-factor model. **μ**, means of each latent factor within subgroup; **ψ**, residuals; **λ**, factor loadings.

Before using SEM trees to determine the most significant predictors, we compared the fits of the two confirmatory factor analysis models (Figure 1) to determine the temperate model of the SEM trees and forests. The one-factor model showed a poor fit for the dataset. The two-factor model achieved good fit on CFI and SRMR but only acceptable fit on RMSEA (Table 1). It should be noted that some level of misfit must initially exist to use the SEM Trees algorithm. Therefore, we set the two-factor model as the temperate model for the SEM trees and forests, attempting to improve the fit of the model based on the assumption that there is heterogeneity among participants.

**Table 1:** Model fit of competing measurement models.

	**χ^2^(df)**	**RMSEA**	**CFI**	**SRMR**
One-factor model	2828.394(20)^***^	0.120 [0.116–0.124]	0.887	0.062
Two-factor model	673.836(20)^***^	0.059 [0.056–0.063]	0.974	0.028

*See Figure 1 for the configuration of the different models*.

*** *p < 0.001; RMSEA, root mean square error of approximation; CFI, comparative fit index; SRMR, standardized root mean square residual*.

### Results of SEM Trees

#### Heterogeneity of Students

Before running the SEM tree analysis, an initial common factor model utilizing eight items and two latent factors were conducted as a homogeneity model. The fits of the factor model and SEM trees were compared on the basis of −2 log likelihood fit function (-2LL) as well as information criteria. Both SEM trees fit more effectively than the model that assumed homogeneity (Table 2). Moreover, the complete SEM tree fit more effectively than the demographic SEM tree across all indices of fit, which indicated that covariates selected by the complete SEM tree provided more information regarding population heterogeneity.

**Table 2:** Fit indexes for factor model and SEM trees (*N* = 9,769).

	**Number of groups**	**Number of parameters**	**-2LL**	**AIC**	**BIC**	**aBIC**
CFM (1-Class)	1	25	123340.91	123390.91	123570.58	123491.14
Demographic SEM tree	5	65	122274.69	122404.69	122871.85	122665.30
Complete SEM tree	6	78	118902.43	119058.43	119499.58	119293.02

*LL, log-likelihood; BIC, Bayesian information criterion; AIC, Akaike information criterion; aBIC, sample-size-adjusted BIC*.

#### Demographic SEM Tree

**Figure 2:**
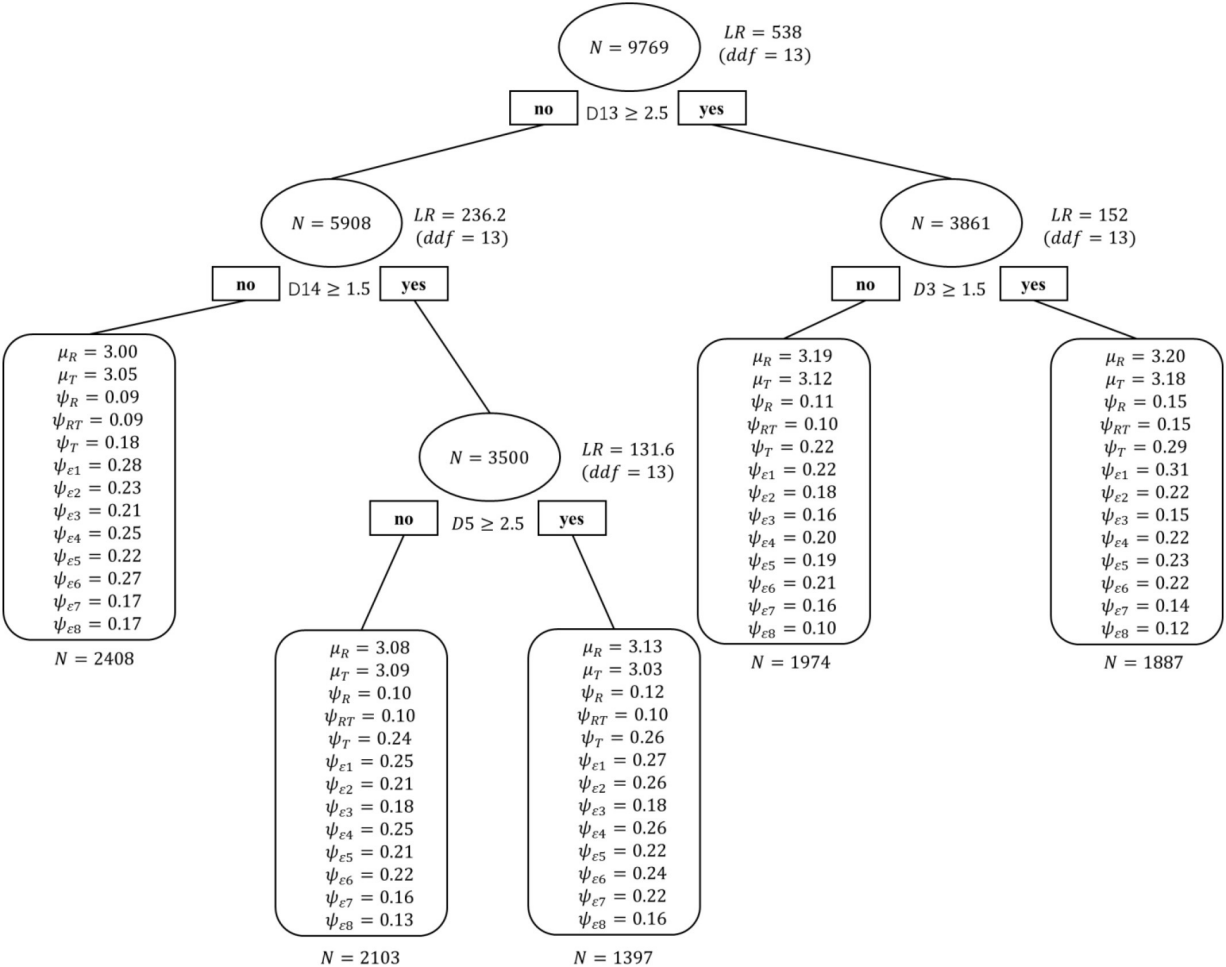
Demographic SEM Tree for students' attitudes toward collaboration. *N*, sample size at each split; *LR*, likelihood-ratio statistic; *ddf* , difference in degrees of freedom; D13, home educational resources; D14, home possessions; D5, mother's education; D3, gender. Each parameter label corresponds to the estimate for that group from the two-factor model depicted in Figure 1B.

Seventeen demographic variables were included in the SEM tree analysis with the two-factor model as the template model to determine their priority in terms of the impact on students' attitudes toward collaboration and to explore the heterogeneity of students. The SEM tree algorithm explored a 6-group model. However, the multiple group analyses showed the best fit on a 5-group model, pruning a false positive splitting. As shown in Figure 2, the first and most informative predictor was home educational resources (D13), classifying students (*n* = 9769) into two groups. On the left side, students with below-average home education resources (*n* = 5908) were classified into two groups by the variable of home possessions (D14): students with disadvantaged home possessions (*n* = 2408) and those with non-disadvantaged home possessions (*n* = 3500). Furthermore, the non-disadvantaged students were classified into two groups by mother's education (D5): students whose mother's education level was below high school (*n* = 2103) and students whose mother's education level was high school or above (*n* = 1397). On the right side were the students with above-average home educational resources (*n* = 3861), who were further divided into two groups by gender (D3): female (*n* = 1974) and male (*n* = 1887).

To summarize, four variables were considered to be most significant for sufficiently identifying the heterogeneity of students: home educational resources, home possessions, mother's education, and gender. In the tree structure, there were interactions among these variables. Comprehensively, group 5, boys with above-average home educational resources, scored the highest (3.20 and 3.18). Group 1, students with below-average home educational resources and disadvantaged home possessions, scored the lowest in relationship (3.00), while group 3, students with below-average home educational resources and non-disadvantaged home possessions, scored the lowest in teamwork (3.03).

#### Complete SEM Tree

**Figure 3:**
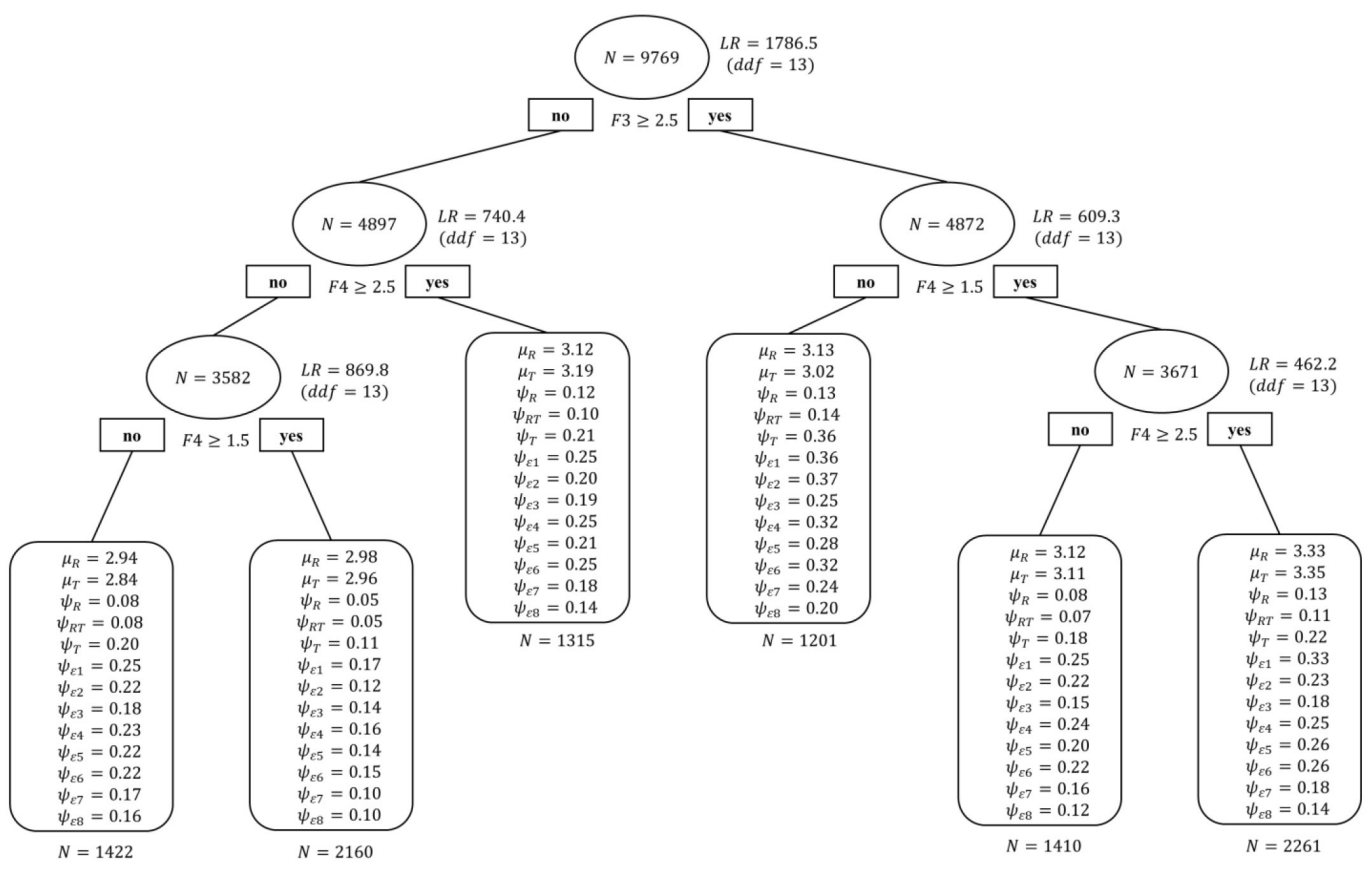
Complete SEM Tree for students' attitudes toward collaboration. *N*, sample size at each split; *LR*, likelihood-ratio statistic; *ddf* , difference in degrees of freedom; F3, achievement motivation; F4, the sense of belonging at school. Each parameter label corresponds to the estimate for that group from the two-factor model depicted in Figure 1B.

For the complete SEM tree analysis, 33 variables were submitted to determine their priority in terms of impact on students' attitudes toward collaboration and to explore the heterogeneity of students. The multiple group analyses showed there was no false positive splitting. As shown in Figure 3, two variables were selected by the SEM tree. The first and most informative split was students' achievement motivation (F3), classifying students (*n* = 9769) into two groups. On the left side, students with below-average achievement motivation (*n* = 4897) were classified into three groups by the sense of belonging at school (F4): students with a low sense of belonging at school (*n* = 1422 on the left), students with a slightly low sense of belonging at school (*n* = 2160 in the middle), and students with an above-average sense of belonging at school (*n* = 1315 on the right). On the right side, students with above-average achievement motivation (*n* = 4872) were also classified into three groups by the sense of belonging at school (F4): students with a low sense of belonging at school (*n* = 1201 on the left), students with a slightly low sense of belonging at school (*n* = 1410 in the middle), and students with an above-average sense of belonging at school (*n* = 2261 on the right).

To summarize, two important variables could help researchers sufficiently identify the heterogeneity of students: achievement motivation and sense of belonging at school. Comprehensively, group 6, students above average in both achievement motivation and sense of belonging at school, scored the highest (3.33 and 3.35), while group 1, students with below-average achievement motivation and a low sense of belonging at school, scored the lowest (2.94 and 2.84).

### *Post-hoc* Group Comparisons

#### Demographic SEM Tree

In support of the demographic SEM tree derived subgroups, significant group differences were found on the indicators of valuing relationship and valuing teamwork, *F*_(8,19464)_ = 57.24, *p* < 0.0001; Wilk's Λ = 0.96, partial η^2^ = 0.023. Means and standard deviations of indicators by SEM Tree-derived subgroups are presented in Table 3, while the *post-hoc* testing is presented in Table 4. All groups significantly differed on valuing relationship, with the exception of group 4 vs. group 5, in which case there was no indication of gender difference on valuing relationship among students with above-average home educational resources. Only two individual comparisons did not significantly differ on valuing teamwork: group 1 vs. group 3 and group 2 vs. group 4. Taken together, *post-hoc* group comparisons support the demographic SEM tree findings. Each group derived by the demographic SEM tree was significantly different from other groups on at least one dimension of students' attitudes toward collaboration.

**Table 3:** Means and standard deviations for study variables by subgroups identified in the demographic SEM tree (*N* = 9738).

**Groups**	**Description**	***n***	**Valuing relationship**		**Valuing teamwork**	
			***M***	***SD***	***M***	***SD***
Group 1	Students with below-average home educational resources and disadvantaged home possessions	2,403	11.94	1.64	12.41	2.01
Group 2	Students with below-average home educational resources, non-disadvantaged home possessions and mother's education was below high school	2,095	12.33	1.63	12.62	2.15
Group 3	Students with below-average home educational resources, non-disadvantaged home possessions and mother's education was high school or above	1,392	12.51	1.78	12.36	2.24
Group 4	Female with above-average home educational resources	1,966	12.81	1.67	12.74	2.06
Group 5	Male with above-average home educational resources	1,882	12.79	1.89	12.97	2.35

*Thirty-one samples with missing data on predictors were not included into the descriptive statistics*.

**Table 4:** Bonferroni *post-hoc* testing across the demographic SEM Tree derived subgroups (*N* = 9738).

**Groups**	**Valuing relationship/Valuing teamwork**			
	**Group 1**	**Group 2**	**Group 3**	**Group 4**
Group 2	−0.39^***^/−0.21^*^			
Group 3	−0.58^***^/0.05	−0.19^*^/0.26^**^		
Group 4	−0.88^***^/−0.33^***^	−0.49^***^/-0.12	−0.3^***^/−0.38^***^	
Group 5	−0.86^***^/−0.56^***^	−0.47^***^/−0.35^***^	−0.28^***^/−0.61^***^	0.02/−0.23^**^

*The numbers represent the difference between the mean score of groups*.

* p < 0.05;

** p < 0.01;

*** *p < 0.001*.

#### Complete SEM Tree

In support of the complete SEM tree derived subgroups, significant group differences were found for the indicators of valuing relationship and valuing teamwork, *F*_(10,19492)_ = 171.96, *p* < 0.0001; Wilk's Λ = 0.84, partial η^2^ = 0.08. Means and standard deviations of indicators by SEM tree derived subgroups are presented in Table 5, while the *post-hoc* testing is presented in Table 6. All groups significantly differed on valuing relationship, with the exception of group 1 vs. group 2 and group 3 vs. group 4 vs. group 5. All groups significantly differed on valuing teamwork. Taken together, *post-hoc* group comparisons support the complete SEM tree findings that individuals belonging to subgroups characterized by a higher level of achievement motivation and/or a higher level of a sense of belonging at school also reported more positive attitudes toward collaboration.

**Table 5:** Means and standard deviations for study variables by subgroups identified in the complete SEM tree (*N* = 9753).

**Groups**	**Description**	***n***	**Valuing relationship**		**Valuing teamwork**	
			***M***	***SD***	***M***	***SD***
Group 1	Students with below-average achievement motivation and low sense of belonging at school	1,419	11.71	1.58	11.58	2.05
Group 2	Students with below-average achievement motivation and slight low sense of belonging at school	2,158	11.86	1.26	12.08	1.53
Group 3	Students with below-average achievement motivation and above-average sense of belonging at school	1,313	12.47	1.76	13.01	2.09
Group 4	Students with above-average achievement motivation and low sense of belonging at school	1,194	12.51	1.93	12.32	2.66
Group 5	Students with above-average achievement motivation and slight low sense of belonging at school	1,408	12.49	1.51	12.72	1.92
Group 6	Students with both above-average achievement motivation and sense of belonging at school	2,261	13.38	1.84	13.68	2.13

*Sixteen samples with missing data on predictors were not included into the descriptive statistics*.

**Table 6:** Bonferroni *post-hoc* testing across the complete SEM Tree derived subgroups (*N* = 9753).

**Groups**	**Valuing relationship/Valuing teamwork**				
	**Group 1**	**Group 2**	**Group 3**	**Group 4**	**Group 5**
Group 2	−0.15/−0.5^***^				
Group 3	−0.76^***^/−1.43^***^	−0.61^***^/-0.93^***^			
Group 4	−0.8^***^/−0.74^***^	−0.65^***^/−0.24^*^	−0.04/0.69^***^		
Group 5	−0.78^***^/−1.14^***^	−0.63^***^/−0.64^***^	−0.02/0.29^**^	0.02/−0.4^***^	
Group 6	−1.67^***^/−2.1^***^	−1.53^***^/−1.6^***^	−0.91^***^/−0.67^***^	−0.87^***^/−1.36^***^	−0.89^***^/−0.96^***^

The numbers represent the difference between the mean score of groups.

* p < 0.05;

** p < 0.01;

*** *p < 0.001*.

### Results of SEM Forests

#### Demographic SEM Forest

**Figure 4:**
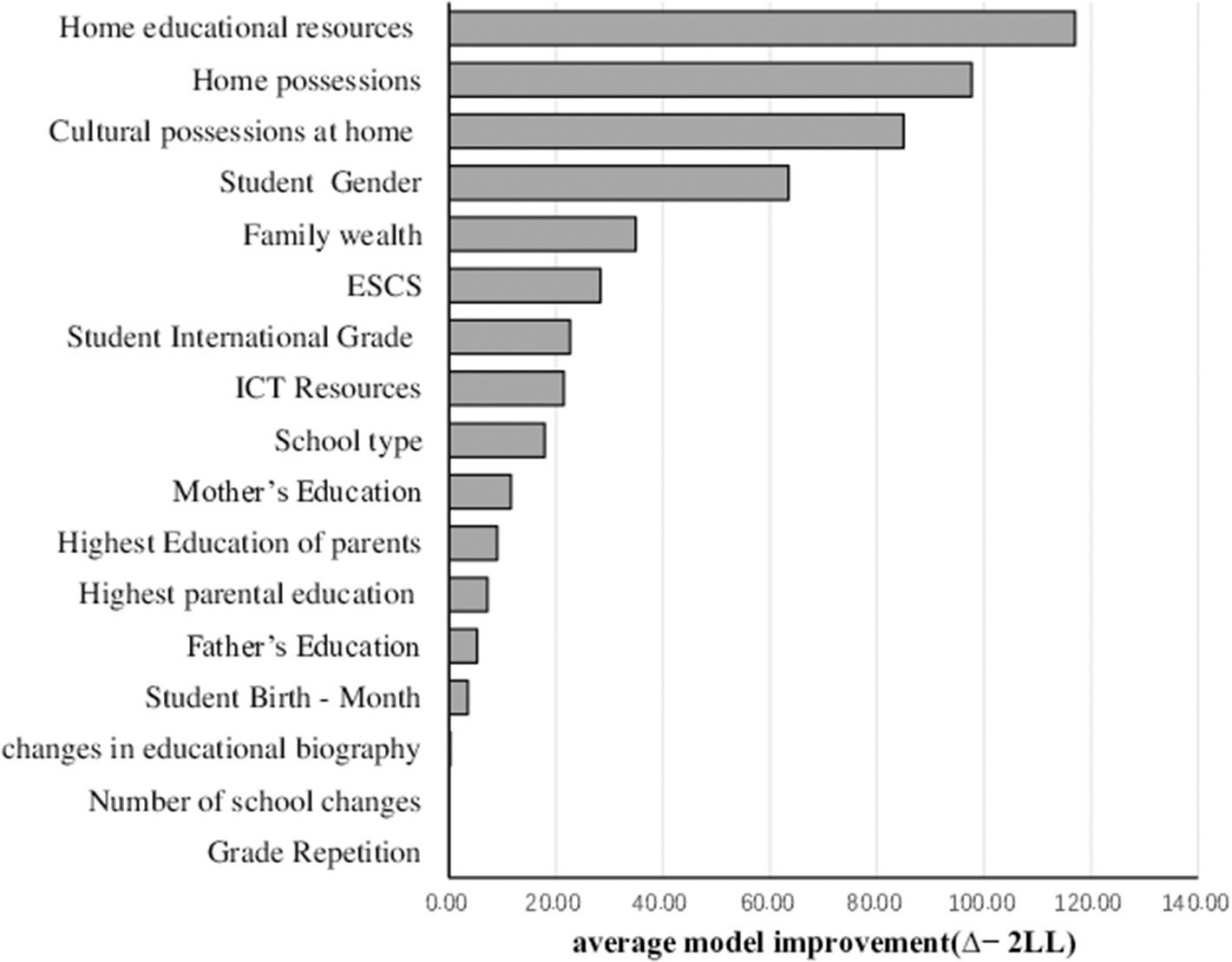
Variable importance of demographic SEM Forest. The x-axis shows the average model improvement (Δ−2*LL*) between the overall model and the two-group model splitting on a covariate. The length of the bar represented the variable importance of each covariable for the factor model, quantified as average increase in model misfit due to randomization.

Demographic variables were listed in descending order of importance in the tree structure according to the split quality (Figure 4). The top variable most improved the model fit (home educational resources), and the bottom variable least improved the model fit (number of school changes). The length of the bar represents the importance of each covariate.

Figure 4 showed concisely that home educational resources, home possessions, and cultural possessions at home were the top three important predictors of students' attitudes toward collaboration. Splitting data based on such covariates could improve the model fit to a large degree. However, grade repetition, number of changes in educational biography, and number of school changes had nearly no influence on the forest model.

#### Complete SEM Forest

**Figure 5:**
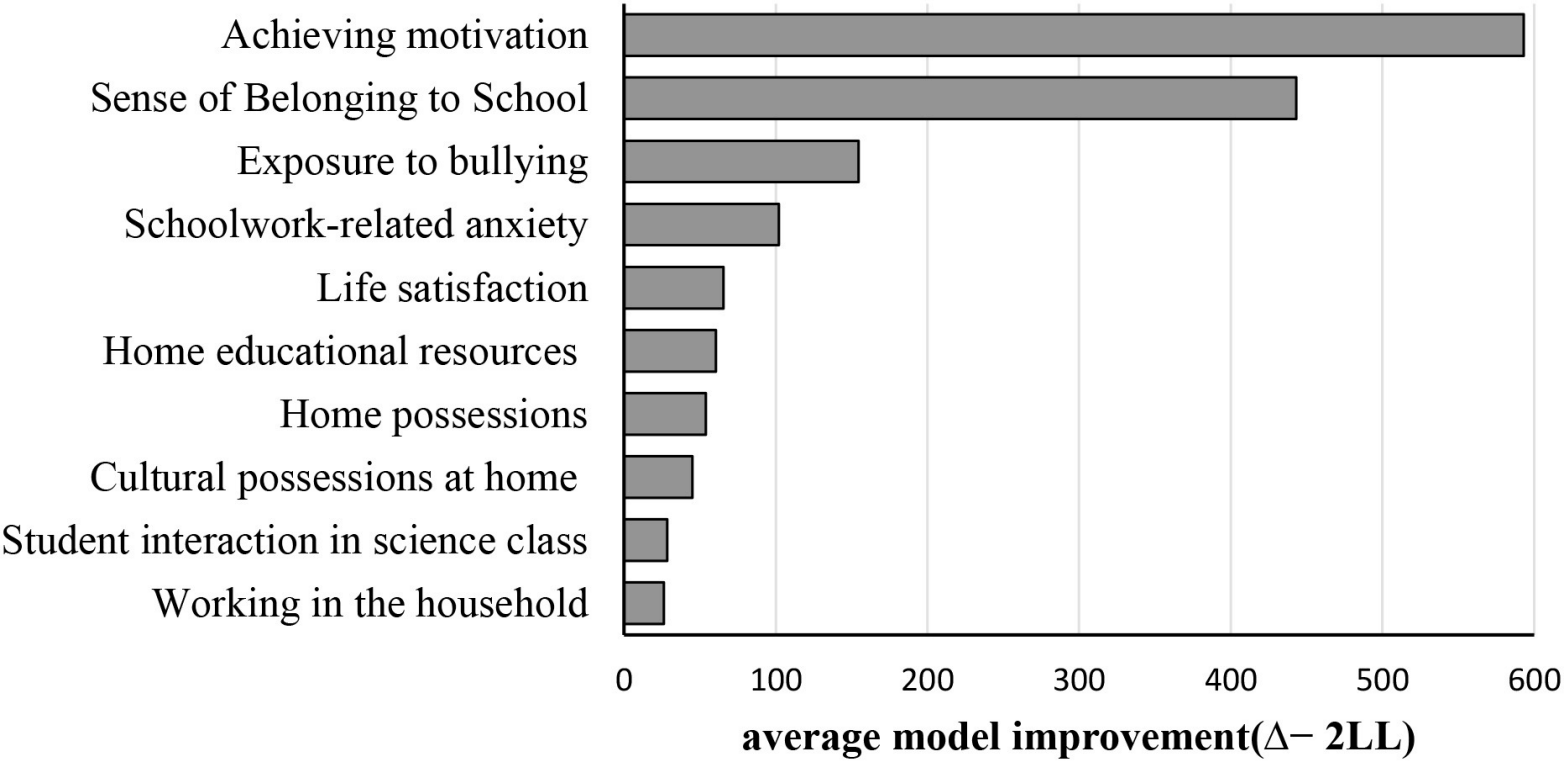
Top 10 variable importance of complete SEM Forest. The x-axis shows the average model improvement (Δ−2*LL*) between the overall model and the two-group model splitting on a covariate. The length of the bar represented the variable importance of each covariable for the factor model, quantified as average increase in model misfit due to randomization.

The top 10 predictive variables were listed in descending order of importance in the tree structure according to the split quality (Figure 5). Achievement motivation and sense of belonging at school were the top two important predictors of students' attitudes toward collaboration and much more important than the other variables. Splitting data based on such covariates could improve the model fit to a large degree.

## Discussion

In the current study, two data mining methods, SEM trees and SEM forests, were used to identify the most predictive variables and their interactions among the associated features and uncover the population heterogeneity regarding students' attitudes toward collaboration in PISA 2015. Seventeen demographic variables as well as 16 variables associated with students' other attitudes and activities were analyzed. In the SEM tree models, home educational resources, home possessions, mother's education, and gender were the most important predictors among the demographic variables, drawing a five-group model, while achievement motivation and sense of belonging at school were the most important variables overall, drawing a six-group model. In addition, the SEM forests provided consistent results with those of the SEM trees, showing that the predictors selected by the tree models ranked the most important in the forest models. These findings have implications for understanding and intervening in students' attitudes toward collaboration.

Among the demographic variables, the first and most important predictor was home educational resources. The results of the SEM trees showed that students with adequate resources may value collaboration, especially in terms of relationship. Moreover, we noticed that the gap among different groups in relationship (the social aspect) was larger than the gap in teamwork (the benefit aspect). For the students with below-average home educational resources, home possession is an important factor to divide different subgroups. The group of students with the least resources (i.e., students with below-average home educational resources and disadvantaged home possessions) scored far lower than other groups and could not be further divided by any other predictors. This may indicate that the extreme lack of resources may severely damage students' attitudes toward collaboration. Scarcity theory describes and explains the psychological consequences of having less than one thinks one needs. A scarcity mind-set captures the attention and reduces cognitive bandwidth, which changes how people think, make their choices, and behave (Mullanathaim and Shafir, 2013). Individuals raised in a stressful, inadequate-resource childhood environment left with less cognitive resources for everything else and may infer that the future is uncertain and surrounding others are unreliable (Samson and Zaleskiewicz, 2019). Individuals with rich material resources have more autonomy and a greater sense of control, so they can afford to maintain an optimistic view of other people, reciprocal interpersonal orientation, and stable interpersonal bonds (Stamos et al., 2019).

The current study goes beyond previous results by considering the interactions of factors and exploring their different influences on different subgroups. For students with medium resources, mother's education, but not father's or parents' education, was selected as the most important and significant predictor. Specifically, students whose mother's education level was below high school reported a significantly higher level of valuing relationship but a lower level of valuing teamwork. That is, students whose mother's education was high school or above may value the relationship with others and perceive themselves as valuable when alone as well as in teamwork. These results again align with previous literature that a lower level of one's mother's education was associated with lower well-being (Sheikh et al., 2016). In China, mothers tend to play a larger role in childrearing. Their parenting skills, or the social communication that takes place in terms of teaching coping skills, have a stronger impact on children's social attitudes. Mothers with higher levels of education might be more knowledgeable about the importance of relationship and more proactive in facilitating students' collaborative attitudes.

Conversely, among students with above-average home educational resources scored the highest in both valuing relationships and valuing teamwork, gender was selected as an important and significant predictor by the SEM tree. Specifically, boys were more likely to highly value teamwork than girls. This gender effect is opposite with PISA 2015 report that Chinese girls were significantly more likely to report that they agree or strongly agree with the four statements that comprise the index of valuing teamwork. One possible explanation is that students with adequate home resources have developed positive values about the social relationship and the trust in others. The teamwork experience further sharps positive attitudes toward collaboration. Comparing with girls, adolescent boys tend to have more team activities, such as team sports and online collaborative games. Positive experience in group activities can improve attitudes toward teamwork (Pop, 2013; Ekimova and Kokurin, 2015). Therefore, among students with above-average family resources, boys value teamwork more highly than girls.

Among all the variables, students' achievement motivation was the most important one. Students with high achievement motivation tend to show more positive collaborative attitudes. From the aspect of information exchange, obtaining goal-relevant information is vital for goal attainment. Individuals with high achievement motivation may tend to obtain more information from their peers (Ryan, 2000). A study of an online collaboration project showed that individuals with higher motivation on career consideration and learning knowledge made a higher contribution to the program (Budhathoki and Haythornthwaite, 2013). It was suggested that those with high achievement motivation tended to spend more time in collaboration and translate the benefits it might bring. However, we should note that mastery and performance goals of achievement breed reciprocity and exploitation orientations in interpersonal communication, respectively (Poortvliet et al., 2007). Mastery goals driven individuals would be more interested in cooperation because such joint efforts could result in higher outcomes than if both actors were to work by themselves (Poortvliet and Giebels, 2012).

The sense of belonging to school was another important variable associated with students' collaborative attitudes. A sense of belonging is defined as feeling accepted and liked by the rest of the group, feeling connected to others, and feeling like a member of a community (Baumeister and Leary, 1995). In school, a sense of belonging gives students feelings of security, identity and community, which, in turn, support academic, psychological, and social development (OECD, 2017c). Students with a sense of belonging build positive and healthy social relationships. Therefore, they may perform an open and cooperative attitude in the collective. In return, a positive cooperative experience would enhance their sense of belonging. McGinn et al. (2005) designed a narrative inquiry to advance theoretical understandings of the notions of collaboration, belonging, and ethical research practices. It was suggested that contemplating the stories of the research team has helped the members to conceptualize collaboration, belonging, and ethical research practice. Currently, the causal direction of effects between the sense of belonging and attitudes toward collaboration remains unclear. However, it is clear that both are fostered through positive interpersonal engagement and experiences.

### Implications and Suggestions

Education is a complex system and various factors have been proved to influence students' attitudes toward collaboration. Specific studies of the relationships between these factors and collaborative attitudes are essential to understand the underlying mechanism. However, for educators, data mining from a large number of potential predictors may provide a complementary way to target “at-risk” populations and develop specific intervention policies with greater effectiveness and lower cost. As mentioned above, the most important predictors of students' attitudes toward collaboration and the corresponding subgroups were uncovered by two data mining methods. Further, we demonstrated the underlying mechanism of their influences. Below are some suggestions for applying these results.

#### Concern for Students With Inadequate Resources

Individuals with stressful and inadequate resources in childhood may perceive that the future is uncertain and others around them are untrustworthy. An extreme lack of resources may severely damage students' attitudes toward collaboration. Therefore, it is critical to provide supportive environments to students with inadequate resources, particularly to develop their sense of control and certainty of the future. Specifically, acknowledging their contribution to the collective and praising their progress are beneficial to cultivate students' sense of control and collaborative attitudes. Besides, for students with medium family resources, attention should be paid to the mothers' influence in the childrearing process.

#### Attach Importance to Intrinsic Achievement Motivation

Achievement motivation facilitates students to exchange information from their peers. Because of the characteristics of students' social communication, peers are one of their main sources of knowledge information. Evidence from achievement goal research suggests that teachers play a central role in fostering student' goals (Darnon et al., 2009). Specifically, teachers should pay more attention to cultivating students' mastery goals which promote students' interest in cooperation.

#### Cultivate the Sense of Belonging at School

Students who feel accepted by the group are more likely to show collaborative attitudes. Teachers should identify students who are feeling socially isolated and organize effective instructions. Freeman et al. (2007) concluded that students' sense of belonging might be fostered in settings characterized by effective instruction, including an emphasis on mastery of meaningful content; warm, respectful interactions between instructor and students; cooperative interactions among students; and smooth organization.

Methodologically, in this study, SEM trees and forests were applied to analyze large-scale educational datasets from a person-centered aspect, which successfully moved beyond what PISA reported. To our knowledge, this is the first study to use these methods on educational cross-sectional data. With ever-increasing quantities of educational data and widespread availability of vast computational resources, this study demonstrates how to use data mining methods to complement other forms of education administration research. Within the DDDM framework, this study illustrates how SEM trees and forests assist educators in improving intervention efficiency. Such methods require less prior information for researchers and explore multivariate data automatically, which might be a future research direction for large-scale education data. We believe that administrators and researchers can benefit from data mining techniques to glean formerly “invisible” information about students and to greatly augment existing understandings of educational phenomena. What's more, its interpretable tree structure rules are useful for setting up early warning systems, especially in the fields of “high-risk” topics like school dropout (Sorensen, 2019) and self-injury (Ammerman et al., 2020).

### Limitations and Future Studies

The strengths of the current study include the use of the comprehensive large-scale educational dataset with a mass of known related factors, the use of SEM trees and forests to determine the most important predictors and corresponding splitting rules to subgroup students, and our suggestions for educators to formulate corresponding interventions. Despite these potential positive features of our research, there are clearly some limitations that should be noted.

The first limitation of this study is the interpretation of the results. Since the current study is cross-sectional and data-driven model can only derive correlations instead of causal relations, the results of such models should be carefully interpreted with classical psychological theories. We would have to conduct a different kind of study to establish the stronger causal claim between these structures and attitudes toward collaboration. Future studies may focus on educational experiments or longitudinal studies to examine the causality. Besides, this study used data from PISA 2015 B-S-J-G (China) only. We should not ignore that subgroups in each country/economy may respond differently. Predictor measures included in this analysis—though relatively comprehensive for an educational dataset—still provide a limited view of the full landscape of relevant student characteristics. Therefore, the result of this study should be carefully generalized.

The second limitation is about the data mining techniques. We suggest that SEM trees and forests might be preferable when analyzing high dimension data, such as datasets containing variables about various aspects of student development. However, we should also note that such modern techniques, as well as DDDM studies, are complementary to, instead of better than, traditional models and educational studies. In addition, tree structures are inherently unstable given random fluctuations in sampling variability. The importance of one split, vs. a different split that was not chosen for the tree structure, may only reflect small improvements in the fit of the model (Ammerman et al., 2019). Therefore, the comprehensive interpretation of SEM trees and forests needs to be considered.

## Conclusion

Using a comprehensive large-scale education dataset, the current study applied SEM trees and forests to elucidate the most important factors and their interactions for students' attitudes toward collaboration from a person-centered aspect. By catching a few key factors, educators could make interventions more effectively and sufficiently. It complemented the absence of previous literature in this field. It was found that family resources were the most important predictors among the demographic variables, which enhanced the scarcity theory that individuals raised in a stressful, inadequate-resource environment left with fewer cognitive resources for positive interpersonal relationship. Interesting interactions were found between family resources and mother's education as well as gender. For students with medium family resources, the higher mother's education predictor higher collaborative attitudes. For students with adequate family resources, there is a slight gender difference in valuing teamwork. On the other hand, achievement motivation and sense of belonging at school were the most important variables overall. Attitudes toward collaboration are defined as how they value the benefit and relationship in collaboration. From the aspect of information exchange, students with high achievement motivation may positively involve in collaboration to translate the benefits it might bring. The sense of belonging reflects individuals' feelings about interpersonal relationship. We suggest educators to concern for students with inadequate resources, attach importance to intrinsic achievement motivation, and cultivate students' sense of belonging at school so as to help students better adapt to the contemporary needs for the ability and attitude of collaboration.

## Data Availability Statement

Publicly available datasets were analyzed in this study. This data can be found here: http://www.oecd.org/pisa/data/2015database/.

## Ethics Statement

Ethical review and approval was not required for the study on human participants in accordance with the local legislation and institutional requirements. Written informed consent from the participants' legal guardian/next of kin was not required to participate in this study in accordance with the national legislation and the institutional requirements.

## Author Contributions

JL and MZ conceived of the study. JL performed the statistical analysis and wrote the manuscript. YL and WS contributed to the literature review. FH contributed to the drafting and revision of the manuscript. MZ supervised the data processing and manuscript writing. All authors read and approved the submitted version.

## Conflict of Interest

The authors declare that the research was conducted in the absence of any commercial or financial relationships that could be construed as a potential conflict of interest.
